# Surface freshening in the subpolar North Atlantic sustaining the weakened AMOC during the late Younger Dryas

**DOI:** 10.1126/sciadv.adv6220

**Published:** 2026-01-01

**Authors:** Defang You, Ruediger Stein, Gerrit Lohmann, Ahmadreza Masoum, Kirsten Fahl, Junjie Wu, Rebecca Jackson

**Affiliations:** ^1^ Alfred Wegener Institute Helmholtz Centre for Polar and Marine Research, 27568 Bremerhaven, Germany.; ^2^ Faculty of Geosciences and MARUM-Center for Marine Environmental Sciences, University of Bremen, 28359 Bremen, Germany.; ^3^ Frontiers Science Center for Deep Ocean Multispheres and Earth System, Key Laboratory of Marine Chemistry Theory and Technology, Ocean University of China, 266100 Qingdao, China.; ^4^ Faculty of Physics and MARUM-Center for Marine Environmental Sciences, University of Bremen, 28359 Bremen, Germany.

## Abstract

The Younger Dryas (YD) cold event is widely attributed to a disruption of the Atlantic Meridional Overturning Circulation (AMOC), driven by the catastrophic Lake Agassiz flood. While recent studies have pinpointed the source and timing of this meltwater pulse, it remains controversial whether this freshwater event alone could have caused a millennial-scale cold period. Biomarker reconstructions from the Labrador Sea/Baffin Bay reveal an abrupt sea ice decline during the mid-YD, parallel with partial AMOC recovery and enhanced Irminger Current inflow. This warm water incursion may have initiated Heinrich Event 0, likely causing surface freshening in subpolar North Atlantic and subsequently triggering a second AMOC decline during the late YD. Model simulations further support a two-phase AMOC weakening associated with surface freshening during the YD. Our biphasic freshwater injection hypothesis provides fresh insights into the mechanisms driving the YD and underscores the critical role of surface freshening in the subpolar North Atlantic in shaping deglacial abrupt climate changes.

## Teaser

A second freshwater pulse into the Labrador Sea may have prolonged the late-YD cold episode after the initial Lake Agassiz flood.

## INTRODUCTION

The Younger Dryas [YD, 12.9 to 11.7 thousand years before the present (ka B.P.)] is recognized as a distinct millennial-scale cold interval that interrupted the deglacial warming of the last glacial period (*1*, *2*). A leading hypothesis attributes the YD to a catastrophic outburst from proglacial Lake Agassiz, which freshened the subpolar North Atlantic’s surface waters, inhibiting North Atlantic deepwater formation and thereby weakening the Atlantic Meridional Overturning Circulation (AMOC) (*3*–*5*). Proxy evidence and model simulations increasingly indicate that the northwestern outlet via the Mackenzie River might have been the primary drainage route for Lake Agassiz, leading to massive freshwater inflow into the Arctic Ocean and subsequently the North Atlantic via the Fram Strait (*6*–*10*). This influx likely caused surface freshening in the Nordic Seas, weakening the AMOC and initiating the YD (*11*).

Despite numerous studies discussing the volumes, fluxes, and routes of the Lake Agassiz flood event, its duration remains elusive. Given potentially high flow rates, some studies propose that the flooding may have occurred over a few years (*12*–*14*), or even as rapidly as 6 to 9 months, based on peak outburst simulations (*15*). However, reconstructions of surface freshening from the Beaufort Sea indicated by the minimum in oxygen isotope ratios of planktic foraminifera suggest that most of the freshwater transport likely occurred within about 130 years (*8*). Nevertheless, the debate remains as to whether a single freshwater event, lasting from several years to a few hundred years, would be sufficient to sustain a millennial-scale cold event like the YD. Although some model simulations that incorporate a combination of freshwater input, radiative forcing, and changes in atmospheric circulation can reproduce the millennial-scale cold conditions of the YD in agreement with proxy records (*16*), many simulations that consider only freshwater forcing still fail to sustain long-term AMOC weakening: Once freshwater input to the Arctic Ocean or North Atlantic is reduced, the AMOC typically exhibits a rapid recovery (*11*, *17*–*19*). Therefore, an additional freshwater pulse may have been required to maintain the AMOC weakening and the associated millennial-scale cold conditions, as suggested by ice core records and the AMOC reconstruction (*20*, *21*).

To investigate these dynamics, high-resolution records from the North Atlantic are essential for capturing abrupt shifts in sea surface conditions and ocean circulation during the last deglaciation. Sea ice reconstructions from the subpolar North Atlantic provide valuable insights into variations in sea surface properties. Increased freshwater discharge, for instance, likely promoted sea ice expansion during periods of rapid ice sheet retreat (*22*–*24*). This expansion would have limited ocean-atmosphere heat exchange, reduced wind stress, weakened ocean ventilation and ultimately, slowed the AMOC (*25*). Conversely, increased advection of warm Atlantic water may have triggered abrupt sea ice declines, enhancing oceanic heat release and potentially contributing to abrupt Greenland warming (*22*, *26*, *27*).

**Fig. 1.**
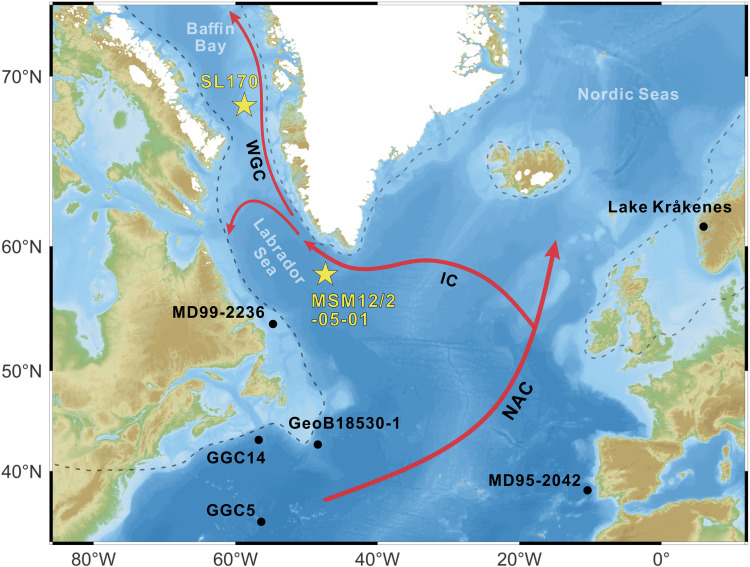
Core sites and warm water inflow of the study area. The yellow stars mark core sites investigated in this study, and black dots indicate other reference core sites. Red arrows show warm surface currents in the study area: North Atlantic Current (NAC), Irminger Current (IC), and Western Greenland Current (WGC). Gray stippled lines show ice sheet extents during the LGM (*84*). Ocean bathymetry and topography data are derived from GEBCO (*85*). The subpolar North Atlantic, as referred to in this study, encompasses the region between ~45°N and 65°N; the Labrador Sea is considered a subregion within this domain.

High-resolution, biomarker-based sea ice records from eastern Baffin Bay and the eastern Labrador Sea (Fig. 1) reveal detailed shifts in deglacial sea ice variability, likely driven by freshwater discharge and warm Atlantic water inflow associated with changes in AMOC strength. By incorporating a suite of decadal- to century-scale proxy records across the North Atlantic, these reconstructions allow us to track changes in sea surface characteristics and AMOC variability throughout the last deglaciation. Our records indicate an abrupt transition during the mid-YD, marked by a distinct reduction in sea ice, partial AMOC recovery, and intensified Irminger Current inflow into the Labrador Sea. This warm water incursion may have triggered the onset of Heinrich Event 0 (HE0), resulting in surface freshening across the subpolar North Atlantic during the late YD, thereby contributing to a weakened AMOC. Simulation from the CLIMBER-X Earth system model support this interpretation, showing a two-phase AMOC decline during the YD, coinciding with simulated negative sea surface salinity (SSS) anomalies in the subpolar North Atlantic. Notably, the simulation provides additional evidence that late-YD surface freshening in subpolar regions may have played a key role in modulating AMOC strength and potentially prolonging the cold conditions of the YD.

## RESULTS AND DISCUSSION

### Biomarker records and sea ice reconstruction

Our biomarker proxy records are derived from data generated in this study from Core SL170 (MSM09/02-0455/13, 68°58.15′ N, 59°23.58′ W; 1078-m water depth) and previously published data from Core MSM12/2-05-01 (57°32.3′ N, 48°44.32′ W; 3491-m water depth) (*24*) (Fig. 1). To reconstruct sea ice conditions, we utilize IP_25_, a well-established biomarker proxy for sea ice algae productivity (*28*, *29*). Given that both permanent ice cover and ice-free conditions can lead to the absence of sea ice algae, and thus no IP_25_ signal, we combine open-water phytoplankton biomarkers, such as dinosterol, brassicasterol, and highly branched isoprenoid III (Z) [HBI-III (Z)], with IP_25_ to distinguish various sea ice scenarios (*30*, *31*). In this study, the P_D_IP_25_ index, calculated from IP_25_ and dinosterol, serves as a key indicator of sea ice variations.

**Fig. 2.**
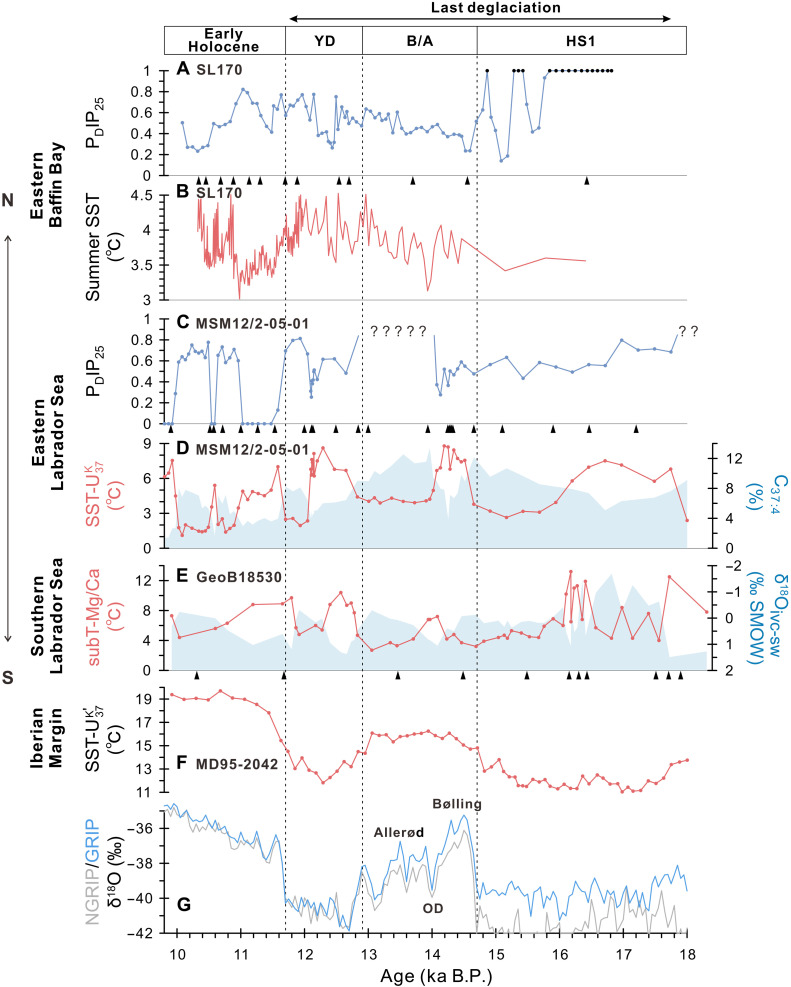
Deglacial changes in sea (sub-)surface characteristics in the polar/subpolar North Atlantic. (**A** and **B**) Proxy records from Core SL170: (A) P_D_IP_25_ values based on dinosterol as proxy for sea ice cover. For the intervals characterized by zero or minimum concentrations of IP_25_ and dinosterol (fig. S1), P_D_IP_25_ values are set to 1 (black circles), assuming permanent sea ice conditions (*30*). (B) Summer sea surface temperature (SST) based on diatom assemblages (*42*). (**C** and **D**) Proxy records from Core MSM12/2-05-01: (C) P_D_IP_25_ values for sea ice cover. The question marks indicate the interval that P_D_IP_25_ values are indeterminable due to extremely low of IP_25_ and dinosterol concentrations (fig. S2), while P_D_IP_25_ values cannot be set to 1 in this case as SSTs are above 0. (D) SST reconstruction based on U^K^_37_ (line) and percentages of C_37:4_ (shading) as proxy for meltwater discharge. (**E**) Subsurface temperature reconstruction based on Mg/Ca ratios of *Neogloboquadrina pachyderma* (sin.) (line) and ice-volume–corrected oxygen isotopic composition of seawater (shading) indicating salinity variations from Core GeoB18530 (*35*). (**F**) SST reconstruction based on ${\text{U}}_{37}^{\text{K}\prime }$ from Core MD95-2042 (*37*). (**G**) δ^18^O values from Greenland Ice Cores (NGRIP and GRIP) (*41*); OD, Older Dryas. Black triangles mark AMS^14^C dates, and the age model of Core GeoB18530 was recalculated in this study. Biomarker records from Core MSM12/2-05-01 have been presented in You *et al.* (*24*).

The P_D_IP_25_ values from Core SL170 are indeterminable during most of Henrich Stadial 1 (HS1) due to the absence or minimum concentrations of IP_25_ and dinosterol (Fig. 2A and fig. S1; see Methods). Following the approach of Müller *et al.* (*30*), P_D_IP_25_ values in such cases are set to “1,” indicating potentially permanent sea ice conditions in eastern Baffin Bay that may have inhibited both sea ice algae production and open-water phytoplankton growth. Such permanent sea ice conditions are terminated with a marked reduction in sea ice at the early Bølling warming period, as indicated by a drop in P_D_IP_25_ to approximately 0.2. From the subsequent Bølling/Allerød period into the onset of early Holocene, the P_D_IP_25_ values show a shift from seasonal sea ice (values near 0.2) to more extensive sea ice coverage (values near 0.8). This phase is followed by a gradual decline in sea ice cover, returning to predominantly seasonal sea ice conditions during the subsequent early Holocene. Moreover, P_D_IP_25_ reconstructions from surface sediments show that modern values in the study area are approximately 0.8 (*32*), suggesting enhanced sea ice formation in the eastern Baffin Bay at present.

Several notable and abrupt sea ice reduction events are superimposed on this long-term trend, each characterized by a rapid drop in P_D_IP_25_ values (ranging from ~0.4 to 0.8), followed by relatively quick recoveries to pre-event levels. The distinct sea ice declines are observed at 15.8, 15.2, and 14.8 ka B.P., relative to the preceding period of potentially permanent sea ice cover during HS1. Another pronounced decline is recorded during the mid-YD, around 12.5 ka B.P., followed by a steady increase in P_D_IP_25_ values from the late YD into the onset of the early Holocene. A further marked decline occurs at approximately 11.6 ka B.P. Such rapid reductions and rebounds may point to a short-lived but intense external forcing.

In contrast, P_D_IP_25_ values from Core MSM12/2-05-01 exhibit little variability from HS1 through the Bølling warming period, remaining consistently around 0.5, which suggests persistent marginal (to seasonal) sea ice cover in the eastern Labrador Sea (Fig. 2C). For the subsequent Allerød period, P_D_IP_25_ values could not be calculated due to extremely low concentrations of both IP_25_ and dinosterol (fig. S2). However, interpreting this scenario as evidence for permanent sea ice cover is unrealistic in this case, as sea surface temperatures (SSTs) reconstructed from alkenone data are well above 0°C during this time interval (Fig. 2D). A more plausible explanation might be that oligotrophic conditions, likely driven by substantial meltwater input during the Allerød period, suppressed primary productivity. During the YD, the P_D_IP_25_ values indicate a range from seasonal sea ice cover (values near 0.3) to extensive sea ice cover (values near 0.8). At the onset of the early Holocene, however, sea ice appears to have been largely absent from the Labrador Sea, followed by two abrupt increases in sea ice formation between 11 and 10 ka B.P. Despite these fluctuations, P_D_IP_25_ reconstructions indicate that sea ice cover has been absent in the eastern Labrador Sea from the mid-Holocene through to modern times (*24*, *32*).

A key characteristic of sea ice variability in Core MSM12/2-05-01 is the occurrence of several abrupt and distinct sea ice expansion events, marked by abrupt increases in P_D_IP_25_ values (ranging from ~0.5 to 0.8), followed by rapid returns to seasonal or ice-free conditions. During the YD, two phases of elevated sea ice cover are recorded: an early YD phase (approximately 12.6 to 12.3 ka B.P.) and a late YD phase (approximately 12.1 to 11.7 ka B.P.), separated by a short interval of reduced sea ice around 12.2 ka B.P., corresponding to the mid-YD. In addition, two intervals of abrupt increases in sea ice cover are observed in the early Holocene, around 11 to 10.6 ka B.P. and 10.4 to 10.0 ka B.P. The sea ice records from Core SL170 and Core MSM12/2-05-01 display a similar pattern during the YD, with relatively high sea ice cover in the early and late phases separated by a period of abrupt decline (Fig. 2, A and C).

### Deglacial changes in sea surface characteristics

Millennial-scale abrupt climate changes in the North Atlantic during the last deglaciation are characterized by pronounced variations in meltwater discharge, ocean circulation, and atmospheric temperatures (*2*, *20*, *21*). Meltwater discharge into the polar and subpolar North Atlantic is widely considered a key driver of abrupt cold events, typically associated with surface freshening, a weakened AMOC, and lower atmospheric temperatures. Conversely, reduced freshwater inflow may have facilitated deepwater formation and strengthened the AMOC, ultimately contributing to abrupt warming events (*2*, *22*, *33*, *34*). To better understand these deglacial sea surface changes, we integrated previously published SST and salinity records from Core GeoB18530 in the southern Labrador Sea and Core MD95-2042 from the Iberian Margin (Fig. 1).

Increased sea surface and subsurface temperatures in the Labrador Sea at the onset of HS1 are indicated by alkenone data and Mg/Ca ratios of the planktic foraminifer *Neogloboquadrina pachyderma* (sin.) (*24*, *35*) (Fig. 2, D and E). These warmer conditions are followed by elevated percentages of tetra-unsaturated alkenones (%C_37:4_), a proxy for low-temperature and/or low-salinity conditions linked to meltwater input (*36*), and by reduced ice-volume–corrected δ^18^O_sw_ values of *N. pachyderma* (sin.), which serve as a salinity proxy. This sequence suggests that enhanced inflow of Atlantic water during early HS1 may have triggered massive meltwater discharge into the Labrador Sea, likely linked to the collapse of the Laurentide Ice Sheet (LIS) (*33*). Surface freshening in the subpolar North Atlantic during HS1 might have sustained marginal sea ice cover in the Labrador Sea (Fig. 2C) and contributed to a weakened AMOC (*21*), resulting in lower SSTs along the Iberian Margin (*37*) (Fig. 2F). However, distinct sea ice declines are observed at 15.8, 15.2, and 14.8 ka B.P. within HS1 in eastern Baffin Bay, which differ from the sea ice reconstruction in the Labrador Sea (Fig. 2, A and C). The weakened AMOC and reduced Atlantic water inflow were therefore likely not the main drivers of these abrupt changes in sea ice. Published Ca/Sr data from Core SL170 show reduced values at these times (*38*) (fig. S1), indicating diminished iceberg and meltwater discharge. Reduced freshwater input may have facilitated sea ice decline, while a thinner halocline could have allowed shallower penetration of Atlantic waters, thereby enhancing sea ice melting (*39*).

During the Bølling warming period, both SSTs and subsurface temperatures increase in the subpolar North Atlantic, coinciding with decreased P_D_IP_25_ values in the Labrador Sea and Baffin Bay. This pattern suggests a strong recovery of warm North Atlantic water inflow, likely resulting in the observed decline in sea ice cover (Fig. 2, A and C to F). Concurrently, lower %C_37:4_ values and higher δ^18^O_ivc-sw_ values in planktic foraminifera indicate reduced freshwater input, supporting the hypothesis that diminished freshwater forcing facilitated an abrupt AMOC recovery and rising atmospheric temperatures (*34*). In contrast, during the Allerød period, lower SSTs and subsurface temperatures are observed in the Labrador Sea (Fig. 2, D and E), accompanied by increased detrital carbonate and silicate input (*24*). In Baffin Bay, the lowest reconstructed summer temperatures occurred around 14 ka B.P. (Fig. 2B), coinciding with elevated detrital carbonate input (*38*) (fig. S1). These proxy records point to enhanced iceberg input and meltwater inflow from the northeastern LIS and Greenland Ice Sheet (GrIS), which may have contributed to the Older Dryas (OD) cold event around 14 ka B.P. (*40*, *41*) (Fig. 2G) and the subsequent cooling of surface waters in the Labrador Sea.

Freshwater inflow and (sub-)SSTs in the Labrador Sea exhibit a biphasic evolution across the YD: Reduced freshwater input and elevated (sub-)SSTs characterize the early YD, whereas increased freshwater discharge and cooler conditions prevail in the late YD (Fig. 2, D and E). In contrast, sea ice cover remains extensive in both phases, interrupted only by a rapid decline during the mid-YD, consistent with the pattern observed in eastern Baffin Bay (Fig. 2, A and C). Reconstructed summer SSTs in eastern Baffin Bay show a modest increase during the YD (Fig. 2B), with a mean error estimate of ±1.14°C based on a transfer function derived from a moderate dataset spanning the broader North Atlantic region (*42*). This modest warming may indicate a relative strengthening of Atlantic water inflow and a potential contribution to enhanced GrIS melting. Moreover, the asynchronous SST changes between the Labrador Sea and the Iberian Margin imply that freshwater forcing in the Labrador Sea was likely regionally confined, exerting limited influence on surface conditions at the Iberian Margin (Fig. 2, D and F).

### Mid-YD transition in the subpolar North Atlantic

**Fig. 3.**
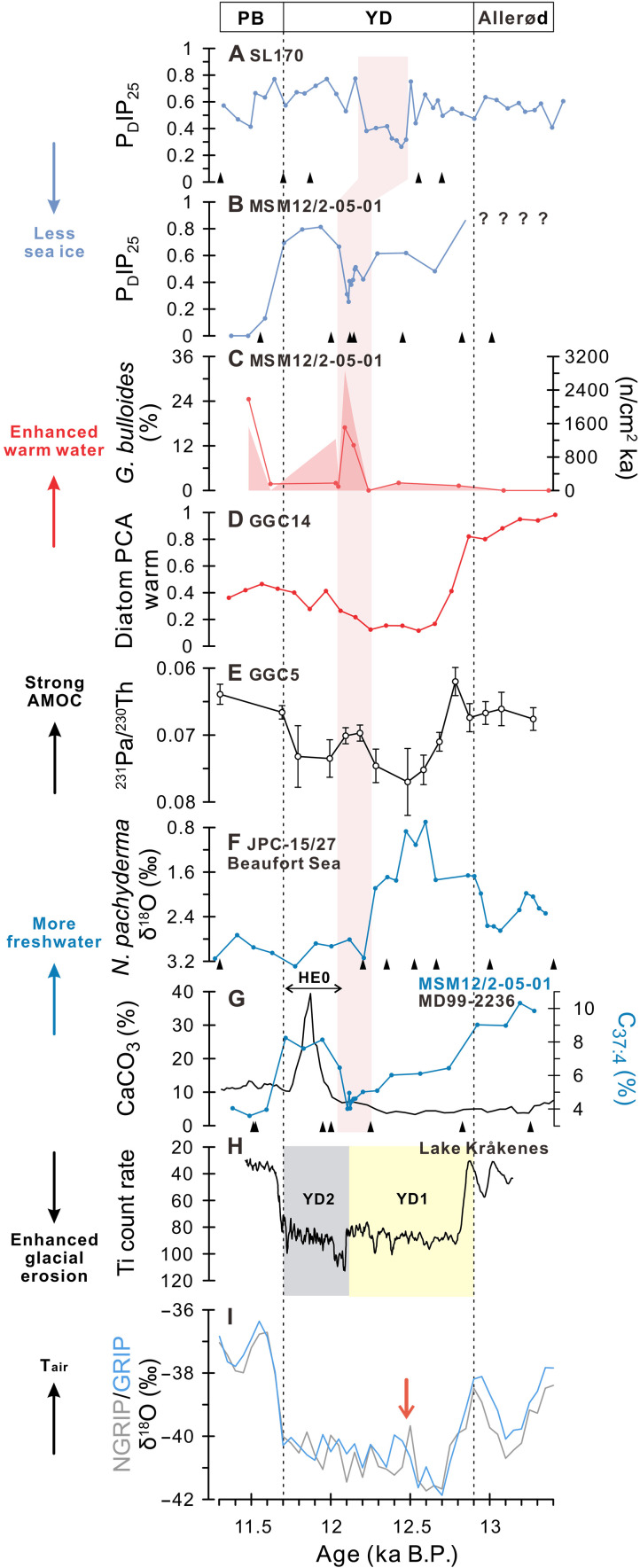
Proxy records of sea surface characteristics, ocean circulation, and detrital input in the North Atlantic during the Younger Dryas. (**A**) P_D_IP_25_ values for sea ice cover from Core SL170. (**B**) P_D_IP_25_ values from Core MSM12/2-05-01. (**C**) Contents (line) and accumulation rates (shading) of *G. bulloides* as proxy for Irminger Current inflow from Core MSM12/2-05-01. (**D**) Factor of diatom assemblage indicating warm Atlantic Water inflow from Core GGC14 (*43*). (**E**) ^231^Pa/^230^Th ratios for the AMOC strength from Core GGC5 (*21*). (**F**) δ^18^O values of planktic foraminifera *N. pachyderma* (sin.) of Core JPC15/27 from Beaufort Sea indicating surface salinity changes (*8*). (**G**) Detrital carbonate contents indicating the collapse of the LIS from Core MD99-2236 (*59*); percentages of C_37:4_ as proxy for meltwater discharge from Core MSM12/2-05-01; black triangles mark recalculated AMS^14^C dates of Core MD99-2236. The age model of Core MD99-2236 was recalculated in this study. (**H**) Titanium count rate indicating glacial erosion of the bedrock surrounding Lake Kråkenes, northwestern Norway; yellow and gray shading highlight the biphasic patterns observed during the Younger Dryas, attributed to reduced freshwater forcing in the Nordic Seas during its later phase (*44*). (**I**) δ^18^O values from Greenland Ice Cores (NGRIP and GRIP) (*41*), red arrow highlights the interval with abrupt rebound in δ^18^O values. Pink shading highlights the abrupt transition during the mid-YD. PB refers to the Preboreal period. Biomarker and foraminiferal records from Core MSM12/2-05-01 have been presented in You *et al.* (*24*, *27*).

Our reconstructions of sea ice and SSTs reveal an abrupt transition in sea surface conditions across the subpolar North Atlantic during the mid-YD, spanning from the Labrador Sea to Baffin Bay. Around 12.2 to 12.1 ka B.P., sea ice cover in the eastern Labrador Sea rapidly declined (Fig. 3B), coinciding with an increased warm Atlantic water inflow via the Irminger Current, as indicated by higher concentrations and accumulation rates of the subpolar warm water planktic foraminifera *Globigerina bulloides* (*27*) (Fig. 3C). A similar reduction in sea ice cover occurred in eastern Baffin Bay around 12.5 to 12.2 ka B.P., likely driven by enhanced warm water incursion during the mid-YD as well (Fig. 3A). The slight temporal offset between these two sea ice records may be attributed to the limited number of age control points for Core SL170 during this interval. Nonetheless, both sites exhibit a comparable and pronounced sea ice decline during the mid-YD. In addition, the principal component analysis–derived factor associated with warm-water diatom species in the western subpolar region shows an upward trend during the mid-YD relative to the lower values of the early YD (Fig. 3D). Although this increase is moderate compared to the peak scores observed during the Allerød period, it still indicates a meaningful increase of warm water influence in this region (*43*).

These findings align with the mid-YD shift proposed by Bakke *et al.* (*44*): In contrast to the stable and colder conditions during the early YD, the late YD was characterized by reduced freshwater forcing in the Nordic Seas, allowing for alternating sea ice cover and Atlantic water inflow. This shift is also supported by the titanium record from Lake Kråkenes in northwestern Norway (Fig. 3H), which reflects enhanced glacial erosion during the late YD, likely linked to reduced freshwater forcing and relatively warmer climate (*44*). In particular, a decadal-resolution δ^18^O record from deep-lake ostracods in Ammersee (southern Germany) reveals a marked spike at the mid-YD transition, interpreted as a short-lived outburst of warm Atlantic waters into the Nordic Seas that led to sea ice melting (*45*). Our reconstruction of sea surface conditions in the subpolar North Atlantic further supports these interpretations.

Although Greenland ice core records depict the YD as a millennial-scale cold phase, it is notable that the extremely cold conditions of the early YD were followed by a partial rebound in temperatures during the mid-YD (*41*) (Figs. 2G and 3I). A comparable warming pattern is also observed in other European lake and glacier records, suggesting relatively milder conditions after the intensely cold early YD (*46*–*50*). However, this mid-YD transition is less evident in high-resolution proxy records from more distant regions, such as the Antarctic ice cores and Asian speleothems (*51*, *52*). The discrepancy between regional and global records suggests that the mid-YD transition was likely confined to the subpolar and polar North Atlantic, rather than representing a globally synchronous event.

A reduction in freshwater forcing in the North Atlantic during the mid-YD likely increased surface water salinity and density, thereby facilitating the recovery of deepwater formation and contributing to the strengthening of the AMOC. Proxy-based reconstructions indicate a partial recovery of the AMOC, although it remained relatively weaker than interglacial levels (*21*) (Fig. 3E). This recovery was accompanied by an enhanced northward transport of warm Atlantic water during the mid-YD, as indicated by increased concentrations of warm water foraminifera and diatom species in the western subpolar regions, in contrast to the extremely cold conditions of the early YD (Fig. 3, C to E). Moreover, enhanced warm water incursion via the Irminger Current may have induced abrupt reductions in sea ice cover in the Labrador Sea and Baffin Bay (Fig. 3, A and B). Sea ice melting may have released freshwater across broad areas of the subpolar North Atlantic, potentially impeding deepwater formation. However, the concurrent poleward reduction in sea ice cover may also have enhanced surface ocean advection due to stronger wind stress acting on the open ocean (*25*), thereby promoting ocean ventilation and further accelerating sea ice retreat. The recovery of the AMOC and enhanced warm Atlantic inflow during the mid-YD suggest that the magnitude of surface freshening associated with sea ice melting was likely insufficient to suppress AMOC activity or inhibit northward heat transport. Nevertheless, despite these regional ocean-atmosphere changes, including increased warm water inflow and reduced sea ice cover (Fig. 3, A to E), they may not have been sufficient to fully restore an interglacial-like circulation regime or terminate the YD.

A partial recovery of the AMOC during the mid-YD supports the notion that freshwater forcing in the North Atlantic diminished following the early YD (*44*), which may not have persisted over millennial timescales. Supporting this view, a distinct interval of low δ^18^O values in *N. pachyderma* (sin.) (a proxy for enhanced surface freshening) in the Beaufort Sea lasted only several hundred years and ended by the mid-YD (*8*) (Fig. 3F), implying a decline in freshwater export from the Arctic to the North Atlantic thereafter. The mid-YD rebound of the AMOC thus suggests that the Lake Agassiz flood alone may have been insufficient to sustain a millennial-scale weakening of the circulation. In line with this, many model simulations that account solely for freshwater forcing typically fail to maintain a prolonged AMOC weakening once freshwater input into the Arctic or North Atlantic is reduced; instead, the AMOC generally recovers rapidly (*11*, *17*–*19*). This further suggests that several hundred years of freshwater discharge may not be sufficient to maintain a weakened AMOC over millennial timescales. However, when additional forcings such as radiative forcing and changes in atmospheric circulation are considered, short-term freshwater pulses into the Mackenzie River outlet may be capable of triggering a millennial-scale cold phase accompanied by a weakened AMOC (*16*). Furthermore, a recent simulation shows that under specific thresholds of freshwater forcing strength and duration, the AMOC can remain in a shutdown state for nearly a thousand years, even without involving additional freshwater sources (*53*). In contrast, cumulative freshwater input from multiple sources may have contributed to sustained surface freshening in the North Atlantic. These include iceberg discharge through Hudson Strait during the late YD (*4*, *54*) and the plausible opening of the eastern outlet of Lake Agassiz at the onset of the YD (*10*, *55*, *56*). Additional potential contributors include freshwater inflow from the North Pacific via the Bering Strait (*57*) and enhanced Arctic sea ice export (*58*).

### Late-YD freshwater inflow and second AMOC weakening

**Fig. 4.**
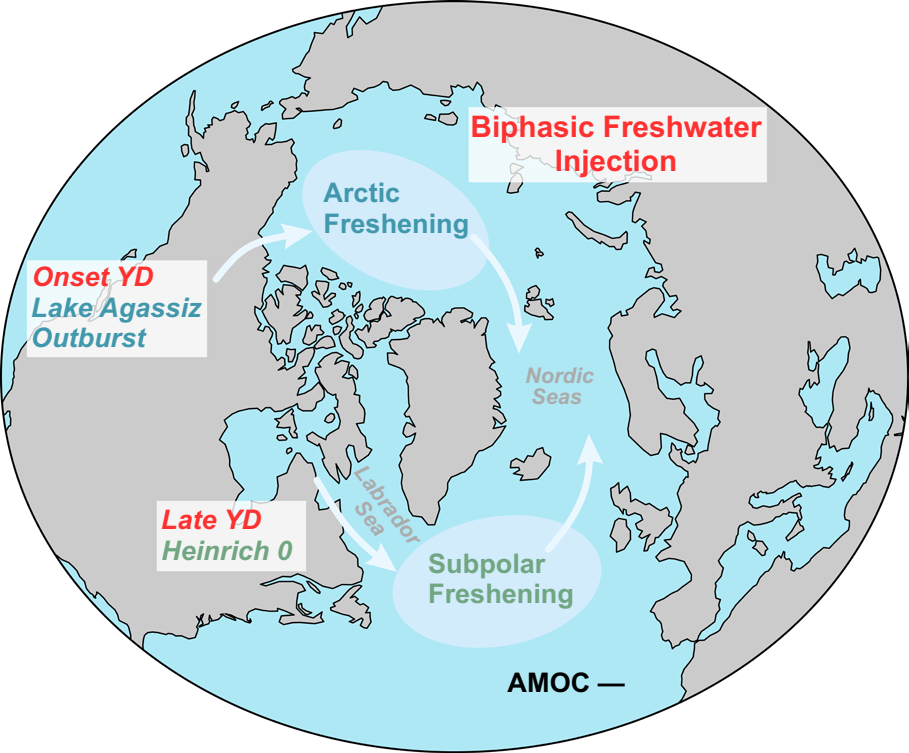
Schematic illustration of the biphasic freshwater injection pattern during the Younger Dryas. White arrows indicate major freshwater transport routes. The biphasic freshwater injection pattern denotes two major freshwater discharges: An initial outburst from Lake Agassiz at the onset of the YD, which may have triggered the cold event, and a subsequent pulse into the Labrador Sea during the late YD, probably associated with HE0, which likely contributed to sustaining a weakened AMOC and the millennial-scale cold episode.

Additional freshwater discharge into the subpolar North Atlantic may have been required to sustain the cold episode during the late YD (Fig. 4). Elevated %C_37:4_ values from the eastern Labrador Sea and lower δ^18^O_ivc-sw_ values from the southern Labrador Sea during this period indicate increased freshwater discharge into the western subpolar North Atlantic, accompanied by abrupt decreases in surface and subsurface temperatures (*24*, *35*) (Fig. 2, D and E). These changes coincide with enhanced detrital carbonate input on the Labrador Shelf (*59*) (Fig. 3G) and across a broader area of the North Atlantic (*54*, *60*, *61*), marking substantial iceberg discharge through the Hudson Strait and a pronounced collapse of the LIS, an event commonly referred to as HE0 (*54*). In addition, elevated detrital carbonate input and higher sedimentation rates in Core SL170 during the YD indicate intensified iceberg and meltwater discharge from the North American-Arctic ice sheets and the western GrIS (*38*) (fig. S1), which may have supplied freshwater to the subpolar regions via Davis Strait.

Most of icebergs from the LIS were likely routed along the western Labrador Sea and transported into the subpolar North Atlantic, where their melting contributed to widespread surface freshening (*62*, *63*). Unlike earlier Heinrich events that involved massive volumes of iceberg discharge, fewer icebergs may have reached the subpolar regions during the YD (*33*, *64*). In addition to iceberg melting, substantial meltwater plumes from the LIS and GrIS may have promoted surface freshening in the Labrador Sea during the late YD (*24*). Nonetheless, this surface freshening likely extended across a broader area of the subpolar North Atlantic, as indicated by elevated detrital carbonate and ice-rafted debris (IRD) input (*54*, *65*), reduced ocean ventilation (*66*), and decreased subsurface temperatures (*67*) in the northeastern Atlantic. Surface freshening may also have led to abrupt increases in sea ice formation in the Labrador Sea and potentially across wider subpolar areas (Fig. 3B). The latent heat effect associated with iceberg melting likely reinforced surface cooling and promoted sea ice expansion, acting as positive feedback that further stabilized upper-ocean stratification (*62*, *68*). This widespread stratification, along with expanded sea ice cover, may have inhibited deepwater formation and thus contributed to a second AMOC decline during the late YD (Figs. 3, E and G, and 4). Model simulations further suggest that freshwater injection into the subpolar North Atlantic has a more pronounced impact on weakening the AMOC compared to other locations (*18*), particularly when deepwater formation regions are directly perturbed, even with relatively small freshwater volumes (*69*).

The occurrence of HE0 may have been triggered by subsurface and surface warming the in Labrador Sea during the mid-YD, driven by intensified Irminger Current inflow (Fig. 3, C and G). This warming may have enhanced basal melting of marine-terminating ice sheets, ultimately leading to the substantial collapse of the LIS associated with HE0 (*27*, *35*). Thus, the abrupt mid-YD transition may have served as a precursor to HE0, initiating massive freshwater discharge into the subpolar North Atlantic and contributing to a subsequent weakening of the AMOC.

### Evidence from paleoclimate simulations

We present a suite of proxy records to reconstruct the history of sea surface conditions in the subpolar North Atlantic during the last deglaciation. Meltwater discharge from the LIS and GrIS likely triggered abrupt sea ice expansion and cooling of SSTs, whereas increased inflow of warm Atlantic water may have led to distinct sea ice decline and increases in SSTs. However, accurately capturing the extent of surface freshening during major meltwater events (e.g., HS1, OD, and YD) remains challenging due to the limited availability of sedimentary records. To address this, we use the climate model CLIMBER-X (*70*) to further explore the relationship between SSS anomalies in the subpolar North Atlantic and AMOC strength during the last deglaciation. Incorporating the recently developed ice-sheet reconstruction PaleoMist as an input (*71*), which prescribes ice sheet evolution and corresponding freshwater flux (fig. S3), CLIMBER-X advances our capacity to simulate climate feedback within the Earth system during this critical period (*72*).

**Fig. 5.**
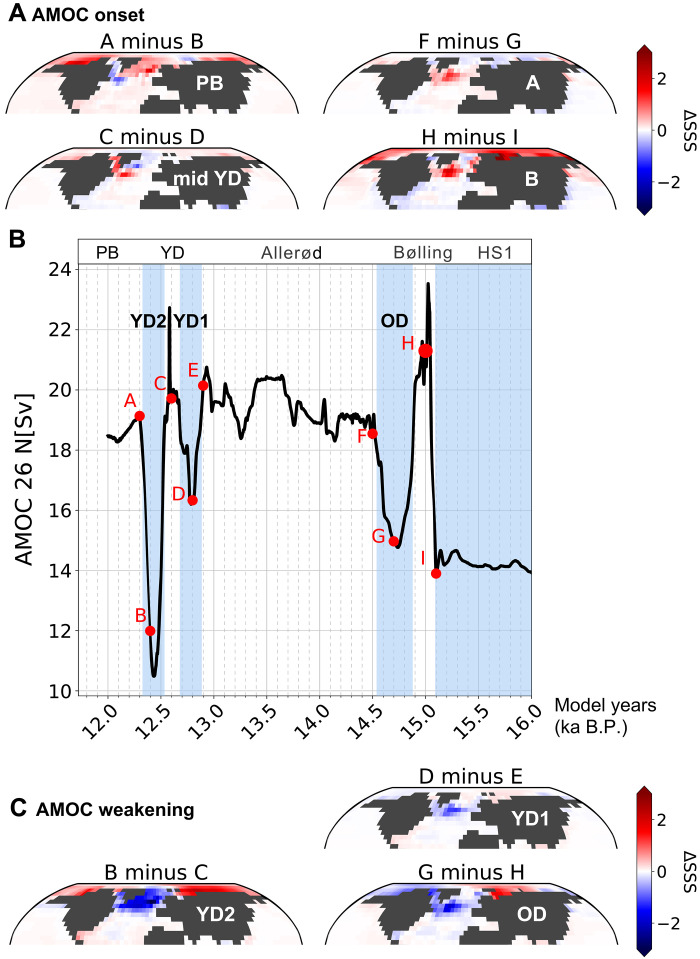
Climate modeling results of deglacial changes in the AMOC and corresponding SSS anomalies in the subpolar North Atlantic. (**A**) Simulated sea surface salinity anomalies (ΔSSS) during the AMOC recovery periods, including the onset of the Preboreal period (PB)/Early Holocene, mid-Younger Dryas (mid-YD), the Allerød period (A), and the Bølling warming period (B). The calculation of ΔSSS is based on the difference in salinity between two selected time slices. (**B**) Simulated changes in the AMOC strength. Red dots mark the selected time slices for SSS calculation. (**C**) Simulated ΔSSS during periods of a weakened AMOC, including the Older Dryas (OD), the early YD (YD1), and the late YD (YD2). Note that the ages shown in the figure represent model ages.

Our simulation reveals a weakened AMOC during HS1, followed by an abrupt recovery during the Bølling warming period (Fig. 5B). Three pronounced declines in the AMOC strength are simulated during the OD and YD. Notably, the YD exhibits a biphasic AMOC decline with a mid-event recovery, broadly resembling the overall pattern inferred from proxy reconstructions (*21*) (Fig. 3E), although the magnitude of the simulated recovery exceeds that observed in the ^231^Pa/^230^Th data. Simulated SSS show negative anomalies in the subpolar North Atlantic during the OD, early YD, and late YD (Fig. 5C), consistent with a weakened AMOC (Fig. 5B) that may result in sea surface cooling in these regions (fig. S4). Notably, these salinity anomalies are mainly concentrated in the western and central subpolar North Atlantic, where the model indicates enhanced freshwater input (fig. S5), due to their proximity to the northern outlet of the LIS (*14*). During the late YD, however, negative SSS anomalies expand across a broader subpolar area, extending into the Nordic Seas. In these regions of reduced SSS, the simulation also shows sea ice expansion and shoaling of the mixed layer (fig. S6), implying a weakened deepwater formation. This reduction contributes to the AMOC weakening, while the expanded sea ice cover may further suppress ocean ventilation, thereby amplifying the effect. These results suggest that, accompanying the collapse of the LIS, freshwater inflow into the Labrador Sea exert a substantial impact on sea surface conditions and ocean circulation in the subpolar North Atlantic. These abrupt declines in SSS and the AMOC are modeled without accounting for the catastrophic Lake Agassiz flood event at the onset of YD. The absence of this superimposed freshwater forcing may explain why negative salinity anomalies are not detected in the Nordic Seas during the early-YD, and why the AMOC weakening is less pronounced than in the late YD, which contrasts with proxy reconstructions (Figs. 3, E and F, and 5B).

During periods of AMOC recovery, such as the Bølling warming period, Allerød period, mid-YD, and Preboreal period, our simulations indicate increased SSS in the subpolar North Atlantic (Fig. 5A). The strongest positive salinity anomalies occur at the onset of the Bølling warming period, consistent with the simulated AMOC overshoot and pronounced sea surface warming in the subpolar regions (Fig. 5B and fig. S4), likely driven by reduced freshwater forcing in the North Atlantic (*21*, *34*). Elevated SSS values in the Labrador Sea and Baffin Bay during the mid-YD align with our proxy reconstructions of sea surface characteristics, indicating reduced surface freshening and enhanced inflow of warm Atlantic water (Figs. 3 and 5A). This mid-YD transition is followed by renewed surface freshening and a second phase of AMOC weakening during the late YD, supporting our interpretation that the warm water incursion during the mid-YD may have triggered the subsequent collapse of the LIS (i.e., the occurrence of HE0), leading to substantial freshwater discharge thereby sustaining the late-YD cold phase. By the end of the YD, negative salinity anomalies are largely confined to western subpolar regions, and freshwater forcing appears to be insufficient to further disrupt large-scale ocean circulation or climate dynamics (Fig. 5, A and B).

In this study, we demonstrate that two distinct freshwater events may have sustained the millennial-scale cooling of the YD. By addressing key uncertainties in the mechanisms regarding the source, magnitude, and duration of freshwater discharge, our findings help bridge the gap between proxy records and model simulations. While Lake Agassiz has long been considered the primary source of freshwater, reconciling the short duration of its outburst flood with the extended cooling observed in ice cores has challenged many model simulations. Our proposed biphasic freshwater injection hypothesis provides an alternative framework for understanding the YD. It resolves existing inconsistencies between proxy records and model simulations and paves the way for future research into the mechanisms of the YD, whether through higher-resolution climate records or refined model simulations.

## MATERIALS AND METHODS

### Sediment materials and chronology

The 683-cm-long sediment core SL170 (MSM09/02-0455/13, 68°58.15′ N, 59°23.58′ W, 1078-m water depth) was retrieved from eastern Baffin Bay during the MSM09/02 Expedition of R/V *Maria S. Merian* (*73*). Age control was established using accelerator mass spectrometry (AMS) ^14^C dates from benthic and planktic foraminifera and mollusc fragments (*38*). A local reservoir correction (△*R* = −49 ± 59) (*74*) and the Marine 20 calibration curve (*75*) were applied to recalibrate the AMS ^14^C dates (table S1). The final age-depth model was generated using the Bacon program (*76*) (fig. S7). The mean sedimentation rate of Core SL170 is 100.5 cm/ka. In this study, we focus on the upper 677 cm of the core, representing 16.82 to 10.09 ka B.P. Within this interval, 95 samples were analyzed for biomarkers, with 21 samples falling within the YD interval. The average temporal resolution of whole record is ~71 years per sample, improving to ~57 years per sample during the YD. Seven data points capture the interval of abrupt P_D_IP_25_ decrease that we interpret as the mid-YD transition. On the basis of Bacon median ages, this event spans 12.47 to 12.22 ka B.P., whereas the 95% confidence interval broadens to 12.68 to 12 ka B.P. Within the YD, four age control points are available based on Bacon median ages, and three age control points remain when considering the 95% confidence range.

The 15-m-long core MSM12/2-05-01 (MSM12/2_647-1, 57°32.31′ N, 48°44.32′ W, 3491-m water depth) was recovered from the Eirik Drift in the eastern Labrador Sea during the MSM12/2 Expedition of R/V *Maria S. Merian* (*77*). Age control was established using AMS ^14^C dates of planktic foraminifera *N. pachyderma* (sin.), calibrated with the Marine20 calibration curve and a global mean reservoir correction (Δ*R* = 0 ± 200) (table S2). Further methodological details are provided in You *et al.* (*24*, *27*). The mean sedimentation rate is 107 cm/ka in the upper 1118 cm, with an assumed rate of 10 cm/ka in the lower 372 cm. In this study, we focus on the interval between 780 and 1174 cm, representing 18 to 9.8 ka B.P. Within this section, 77 samples were analyzed for biomarkers, including 16 samples within the YD. The average temporal resolution is ~106 years per sample for the whole record and ~71 years per sample during the YD. Eight data points capture the abrupt P_D_IP_25_ decrease associated with the mid-YD transition. On the basis of Bacon median ages, this event spans 12.2 to 12.1 ka B.P., whereas the 95% confidence range broadens to 12.5 to 11.73 ka B.P. For this core, we applied a conservative reservoir correction uncertainty of Δ*R* = 0 ± 200 years, which is broader than the Δ*R* = 0 assumption commonly used in subpolar North Atlantic studies. As a result, the estimated duration of the mid-YD transition (95% confidence interval) remains relatively large and should be regarded as approximate. Within the YD, five age control points are available based on Bacon median ages, and four age control points remain when considering the 95% confidence range.

A ^14^C plateau approach, as proposed by Sarnthein *et al.* (*78*), could serve as an additional method for AMS ^14^C calibration. However, identifying and applying such plateaus would require a greater number of AMS ^14^C dates to confirm their validity. Therefore, we did not follow this approach in the current study.

### Biomarker analysis

Five grams of freeze-dried and homogenized sediments were extracted by ultrasonication with dichloromethane/methanol (DCM/MeOH, 2:1 v/v). Internal standards 7-hexylnonadecane (7-HND, 0.076 μg) and 5α-androstan-3β-ol (androstanol, 10.8 μg) were added before extraction. The concentrated extracts were separated into hydrocarbon and sterol fractions by open silica gel column chromatography, using 5 ml of *n*-hexane and 9 ml of ethylacetate/*n*-hexane, respectively. The sterol fraction was derivatized with 200 μl of bis-trimethylsilyl-trifluoracet-amid (60°C, 2 hours) before analysis.

Hydrocarbons and sterols were analyzed by gas chromatography/mass spectrometry using Agilent systems (7890GC-5977A for hydrocarbons and 6850GC-5975A for sterols). HBIs were quantified based on their molecular ions [*m*/*z* 350 for IP_25_, *m*/*z* 348 for HBI-II, and *m*/*z* 346 for HBI-III (Z)] relative to the fragment ion *m*/*z* 266 (7-HND). Brassicasterol (24-methylcholesta-5,22E-dien-3β-ol) and dinosterol (4a-23,24-trimethyl-5a-cholest-22E-en-3β-ol) were quantified as trimethylsilyl ethers, using molecular ions (*m*/*z* 470 for brassicasterol and *m*/*z* 500 for dinosterol) in relation to the molecular ion *m*/*z* 348 of androstanol. External calibration curves and specific response factors were applied to balance the different responses of molecular ions of the analytes and the molecular/fragment ions of the internal standards. Biomarker concentrations were normalized on the basis of total organic carbon content of each sample. For additional analytical details, see Fahl and Stein (*31*).

PIP_25_ values, a semiquantitative index for sea ice reconstruction, were calculated using Eq. 1 (*30*). The index is derived from the seasonal sea ice biomarker IP_25_ and open-water phytoplankton biomarker [e.g., dinosterol, brassicasterol, and HBI-III (Z)]

$${\text{PIP}}_{25}=\left[{\text{IP}}_{25}\right]/\left(\left[{\text{IP}}_{25}\right]+\left[\text{Phytoplankton biomarker}\right]\times c\right)$$ (1)

The balance factor (*c*) corresponds to the ratio of mean concentration of IP_25_ and phytoplankton biomarker. The *c* factor of 0.404 was applied to Core SL170, while a value of 0.044 was used for Core MSM12/2-05-01. In this study, we used P_D_IP_25_ values, based on IP_25_ and dinosterol concentrations (figs. S1 and S2), to indicate sea ice variations.

### Model simulation

The study uses CLIMBER-X, an Earth system model of intermediate complexity (*70*), to simulate climate changes during the last deglaciation. The model operates at a horizontal resolution of 5° × 5° for all components and combines multiple interconnected subsystems: a semi-empirical statistical-dynamical atmosphere model, a three-dimensional frictional-geostrophic ocean model, a dynamic-thermodynamic sea ice component, and a land surface module with dynamic vegetation. Its atmospheric component is based on a statistical-dynamical approach that incorporates several simplifications and assumptions explicitly derived from the present-day climate. This configuration allows for efficient simulation of mean climate states, achieving computational speeds ~1000 times faster than conventional general circulation models under comparable hardware conditions (*70*). The model is especially well-suited for multimillennial paleoclimate experiments, although the coarser spatial resolution may limit the detailed representation of regional processes and freshwater routing.

Greenhouse gas concentrations (CO_2_, N_2_O, and CH_4_) are prescribed by Köhler *et al.* (*79*) while orbital parameters follow Laskar *et al.* (*80*). Deglacial boundary conditions for ice sheets, bathymetry, and land-sea distribution are prescribed using the PaleoMist reconstruction (*71*), which has been shown to be robustly consistent with deglacial scenarios (*72*).

The transient simulation spans the period from the Last Glacial Maximum (LGM; 22 ka B.P.) to the early Holocene (10 ka B.P.). Greenhouse gas concentrations and orbital parameters are updated annually, while changes in topography, bathymetry, and ice sheet distributions are applied at 100-year intervals. Ocean freshwater fluxes are derived from precipitation-evaporation, sea ice fluxes, and runoff. In this study, we did not perform dedicated sensitivity experiments that explicitly test the volume and location of freshwater input. Instead, on the basis of the PaleoMist reconstruction used in this model, prescribed changes in ice volumes were converted into liquid water fluxes (fig. S3), which were routed into the ocean following the steepest surface gradient (fig. S5). Future freshwater sensitivity experiments with higher-resolution climate simulations will be needed to better capture the regional freshwater processes highlighted by our results. Such experiments would also allow for systematic data-model comparisons across key time intervals. Further model details are provided in the Supplementary Materials and in Masoum *et al.* (*72*).

Iceberg dynamics are not explicitly included in the model. Instead, freshwater input from iceberg melt is accounted for through prescribed ice sheet mass loss. Previous studies have shown that iceberg melting contributes to both surface freshening and additional cooling due to latent heat uptake during melting (*81*–*83*), which may promote sea ice formation. Incorporating an explicit iceberg module could amplify salinity anomalies and influence sea ice concentration in the subpolar regions. Nevertheless, the absence of a dynamic iceberg representation is not expected to fundamentally alter the main conclusions of this study.
