# Supplementary material for: Surface freshening in the subpolar North Atlantic sustaining the weakened AMOC during the late Younger Dryas
Source: Sci Adv. 2026 Jan 1;12(1):eadv6220. doi: 10.1126/sciadv.adv6220 (PMC12757042; doi:10.1126/sciadv.adv6220)
Supplement: Supplementary file 1 — Figs. S1 to S7 Tables S1 and S2 Legend for data S1 [file sciadv.adv6220_sm.pdf]

Science Advances
AAAS

Supplementary Materials for

Surface freshening in the subpolar North Atlantic sustaining the weakened AMOC during the late Younger Dryas

Defang You *et al.*

Corresponding author: Defang You, defang.you@geow.uni-heidelberg.de

Sci. Adv. **12**, eadv6220 (2026)
DOI: 10.1126/sciadv.adv6220

The PDF file includes:

Figs. S1 to S7
Tables S1 and S2
Legend for data S1

Other Supplementary Material for this manuscript includes the following:

Data S1

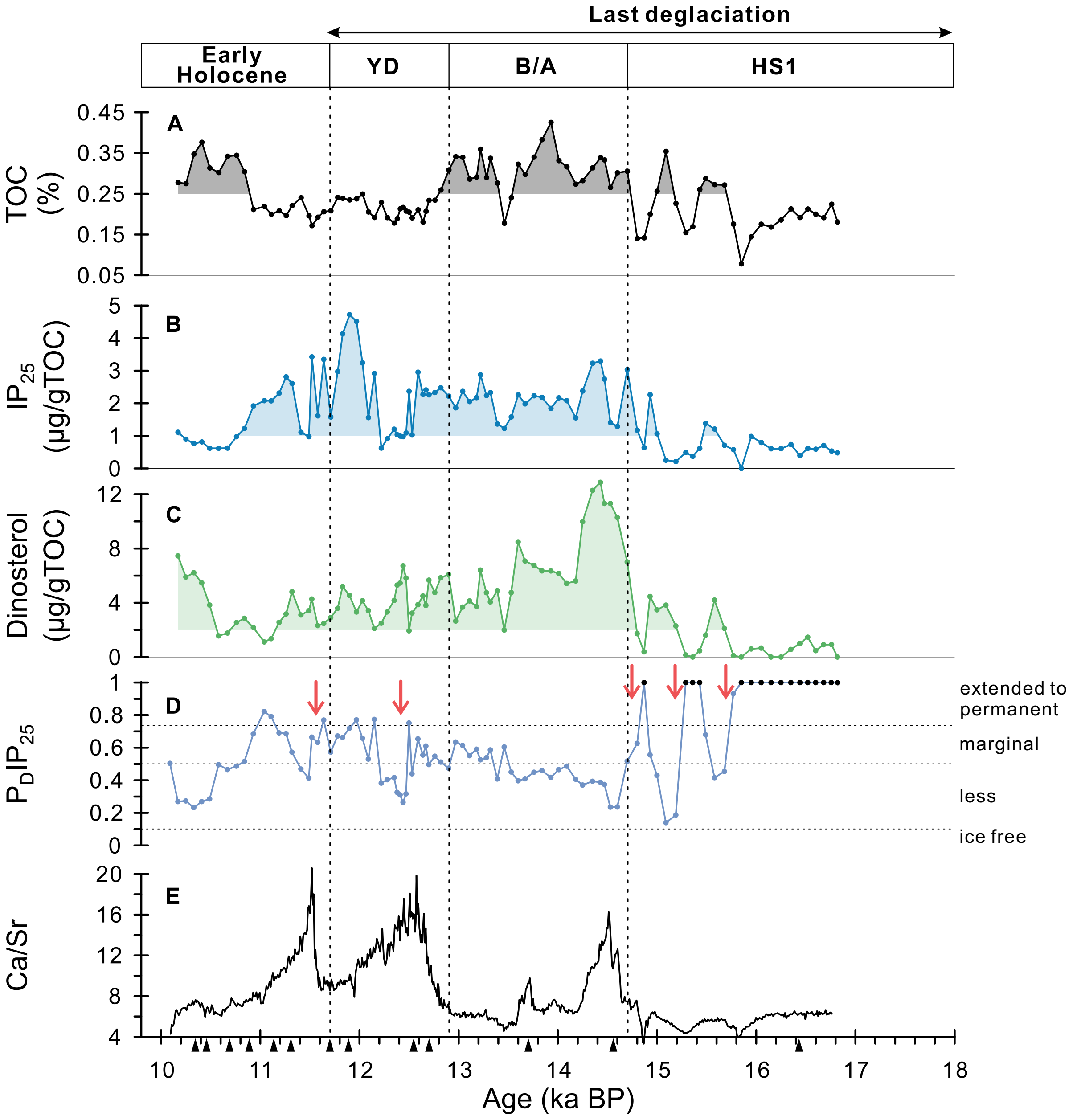

Fig. S1. Concentrations of total organic carbon, biomarkers, and XRF-Ca/Sr ratios of Core SL170.

(**A**) Concentrations of total organic carbon (TOC). (**B**) Concentrations of IP_{25} as proxy for sea-ice algae production. (**C**) Concentrations of dinosterol as proxy for open-water phytoplankton production. (**D**) P_DIP_{25} values based on IP_{25} and dinosterol concentrations as proxy for sea ice cover. For the intervals characterized by zero or minimum concentrations of IP_{25} and phytoplankton biomarkers, P_DIP_{25} values are indeterminable and are set to 1 (black circles), assuming permanent sea ice conditions (*30*). (**E**) Published XRF-Ca/Sr ratios (*38*), indicating detrital carbonate input. The shadings indicate relatively higher TOC and biomarker contents. Red arrows highlight abrupt decreases in P_DIP_{25} values. Black triangles mark AMS^{14}C dates.

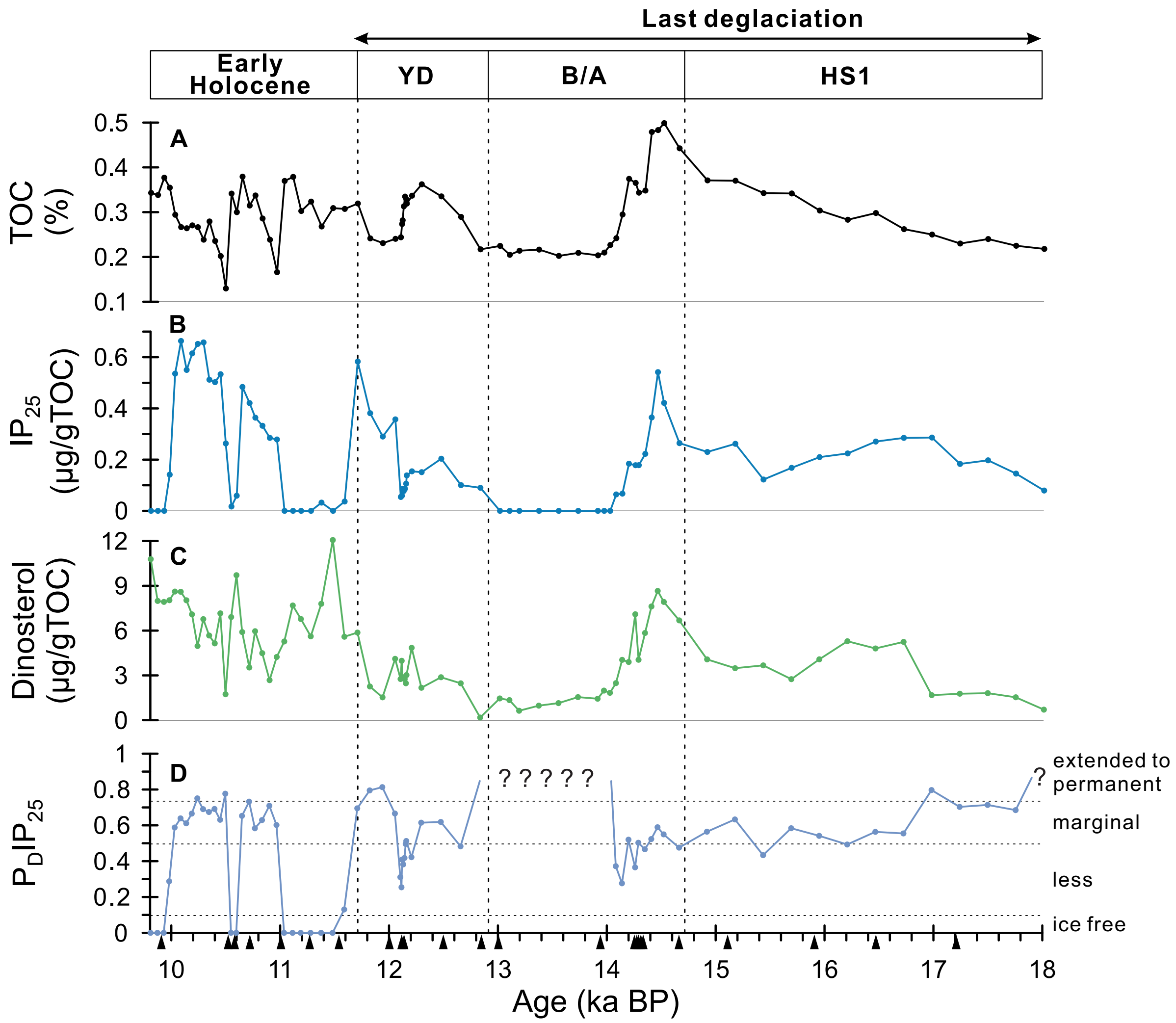

Fig. S2. Concentrations of total organic carbon and biomarkers of Core MSM12/2-05-01. (**A**) Concentrations of total organic carbon (TOC). (**B**) Concentrations of IP_{25} as proxy for sea-ice algae production. (**C**) Concentrations of dinosterol as proxy for open-water phytoplankton production. (**D**) P_DIP_{25} values based on IP_{25} and dinosterol concentrations as proxy for sea ice cover. For the interval with zero or minimum concentrations of IP_{25} and dinosterol, P_DIP_{25} values are indeterminable (highlighted by question marks, see Ref. *30*), while they cannot be set to "1" assuming a permanent sea ice cover in this case, as SST values are well above 0 °C during this time interval. Black triangles mark AMS^{14}C dates.

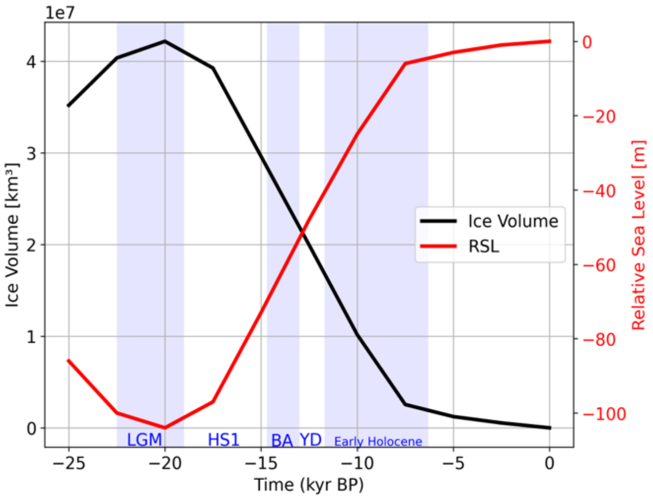

Fig. S3. Changes in ice sheet volumes and relative sea level from PaleoMist reconstruction. This plot illustrates the evolution of ice volume and the corresponding sea-level changes over the past 25 kyr, based on the PaleoMist reconstruction used in the model. Prescribed changes in ice volumes were converted into liquid water fluxes, which were routed into the ocean following the steepest surface gradient.

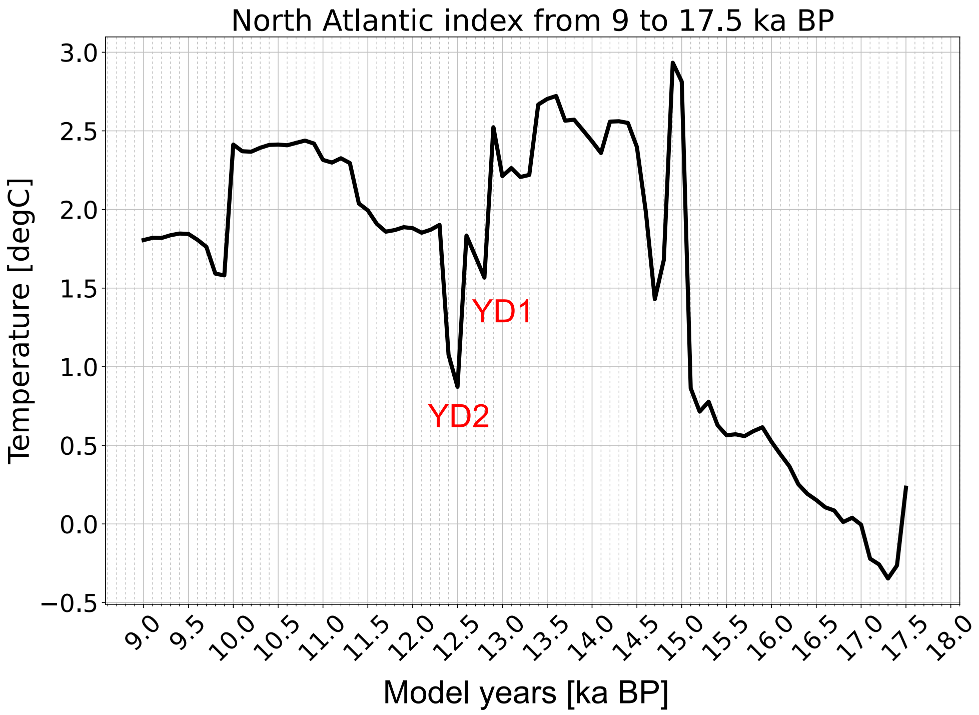

Fig. S4. Simulated (CLIMBER-X) North Atlantic temperature index during the last deglaciation.

The North Atlantic index is defined as the average temperature over 50°N-70°N and 45°W-0°W in the subpolar North Atlantic, capturing the area sensitive to sea surface temperature/salinity changes. The index shows abrupt decreases in temperature in the subpolar North Atlantic during the early Younger Dryas (YD) and the late YD, respectively.

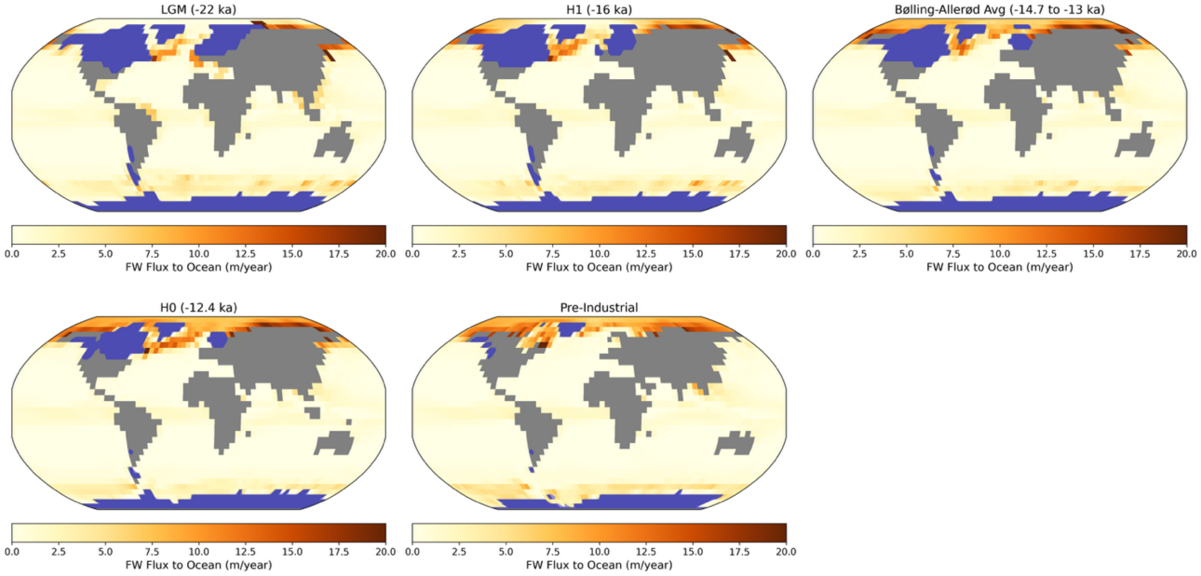

Fig. S5. Freshwater flux into the ocean at five transient time slices.

The freshwater flux to the ocean was calculated based on a combination of precipitation–evaporation balance, land runoff, sea ice fluxes, and changes in ice sheet volumes. This flux was updated daily throughout the transient simulation, with dynamic contributions from each component. All climate model components operated on a shared base time step of one day, which also served as the coupling frequency between model modules. Changes in ice sheet volume represented one of the major sources of freshwater input in the simulation. As expected, the high-latitude Northern Hemisphere exhibits elevated freshwater input, particularly in the subpolar North Atlantic, consistent with the negative sea surface salinity anomalies shown in Figure 5 of the main text. These high-latitude fluxes are primarily driven by substantial ice sheet meltwater discharge.

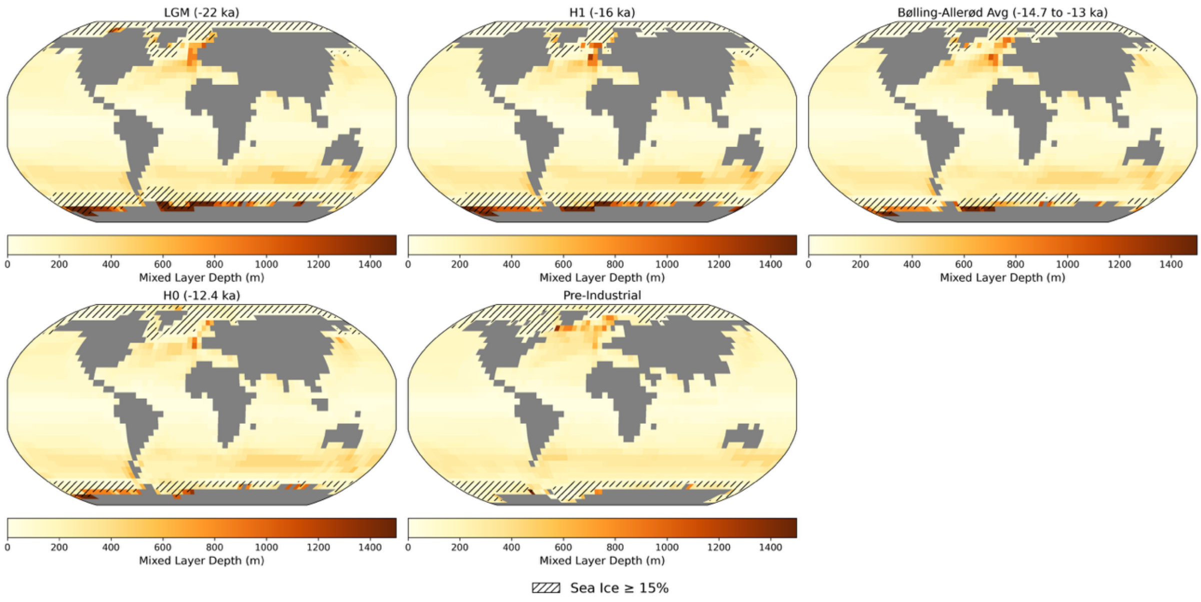

Fig. S6. Mixed layer depth (shading) and sea ice distribution (hatched shading, concentration >15%) at five transient time slices.

The simulated distribution and depth of the ocean mixed layer can serve as an indicator for potential sites of deep-water formation. This plot presents global maps of mixed layer depth at five transient time slices based on CLIMBER-X model simulations. To illustrate the influence of surface freshening and sea ice on deep-water formation, we also include sea ice extent (defined as areas with >15% concentration) in the mixed layer depth plots (hatched shading). These maps clearly show that the presence of sea ice in the North Atlantic is associated with shallower mixed layers, prohibiting the deep-water formation.

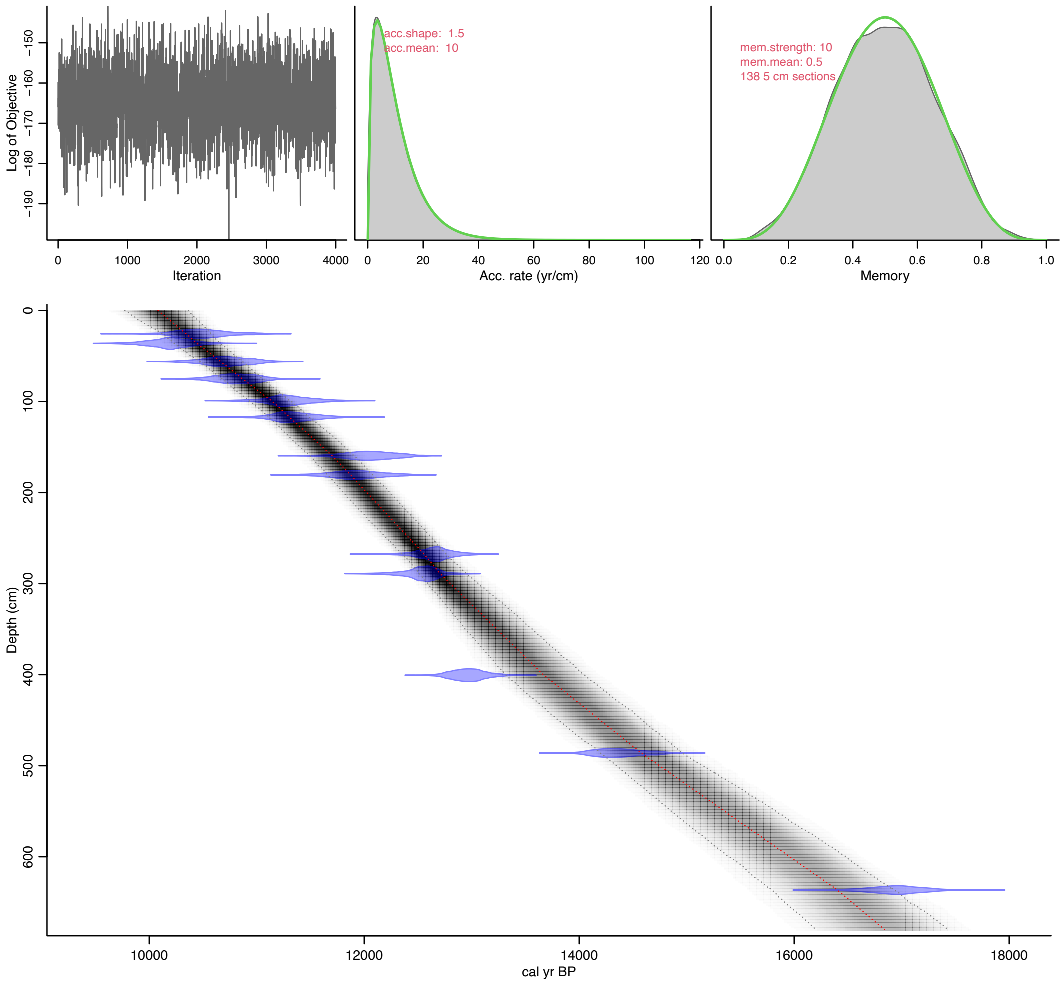

Fig. S7. Age model of Core SL170.

Bayesian age-depth model was established based on AMS^{14}C dates from Jackson et al. (*38*) using the Marine 20 calibration curve (*75*) and Bacon program (*76*). Grey stippled lines indicate 95% confidence intervals of calibration age.

Table S1. Recalculated AMS ^{14}C dates of Core SL170 based on the Marine 20 calibration curve.

Lab ID	Depth (cm)	Species	AMS ^{14}C age (yr.)	Reference	△R	Calib. age median (yr. BP)	Model age median (yr. BP)	Model age_2σ uncertainty (yr.)
ETH-55678	24-27	mixed benthic	9668±112	Jackson et al., (38)	-49±59	10472	10344	227
ETH-55679	35-37	mixed benthic	9460±80	Jackson et al., (38)	-49±59	10201	10444	215
ETH-55680	55-57	mixed benthic	9833±83	Jackson et al., (38)	-49±59	10713	10664	198
ETH-55681	74-76	planktic	9901±82	Jackson et al., (38)	-49±59	10815	10870	190
ETH-55683.1	98-100	mixed benthic	10243±80	Jackson et al., (38)	-49±59	11281	11139	188
ETH-55685	116-118	mixed benthic	10274±86	Jackson et al., (38)	-49±59	11326	11304	188
ETH-58351	159-160	mollusc fragments	10755±85	Jackson et al., (38)	-49±59	12053	11704	205
ETH-58353	180-181	mixed benthic	10671±85	Jackson et al., (38)	-49±59	11918	11874	210
ETH-55687	266-269	mixed benthic	11267±100	Jackson et al., (38)	-49±59	12666	12549	204
ETH-58354	288-290	mixed benthic	11150±75	Jackson et al., (38)	-49±59	12561	12696	218
ETH-55688	399-402	planktic	11597±104	Jackson et al., (38)	-49±59	12966	13672	366
KIA 40766	484-488	planktic	12730±60	Jackson et al., (38)	-49±59	14343	14579	382
ETH-58355	636-637	planktic	14640±130	Jackson et al., (38)	-49±59	16953	16414	570

All AMS^{14}C dates were recalibrated using the Marine 20 calibration curve (*75*) and a recently published local reservoir correction for eastern Baffin Bay (*74*). Modelled ages were obtained with the Bacon program (*76*). The 2σ uncertainties represent the 95% confidence ranges of the modelled ages.

Table S2. AMS ^{14}C dates of Core MSM12/2-05-01 based on the Marine 20 calibration curve.

Lab ID	Depth (cm)	Species	AMS ^{14}C age (yr.)	Reference	△R	Calib. age median (yr. BP)	Model age median (yr. BP)	Model age_2σ uncertainty (yr.)
AWI-5789.1.1	799.5	*N. pachyderma*	9585±117	You et al., (24)	0±200	10305	9920	344
AWI-5793.1.1	919.5	*N. pachyderma*	9831±127	You et al., (24)	0±200	10644	10538	341
AWI-5794.1.1	929.5	*N. pachyderma*	9606±120	You et al., (24)	0±200	10335	10586	333
AWI-5795.1.1	952.5	*N. pachyderma*	9641±119	You et al., (24)	0±200	10384	10715	333
AWI-5798.1.1	999.5	*N. pachyderma*	9860±119	You et al., (24)	0±200	10683	11023	367
AWI-5799.1.1	1029.5	*N. pachyderma*	10029±125	You et al., (24)	0±200	10903	11264	359
AWI-5800.1.1	1059.5	*N. pachyderma*	10428±128	You et al., (24)	0±200	11492	11571	378
AWI-5803.1.1	1108.5	*N. pachyderma*	10543±129	You et al., (24)	0±200	11653	12093	340
AWI-6484.1.1	1114.5	*N. pachyderma*	10621±100	You et al., (24)	0±200	11764	12124	338
AWI-6485.1.1	1118.5	*N. pachyderma*	11130±101	You et al., (24)	0±200	12430	12147	340
AWI-6486.1.1	1123.5	*N. pachyderma*	11141±94	You et al., (24)	0±200	12445	12469	269
AWI-6487.1.1	1127.5	*N. pachyderma*	11752±95	You et al., (24)	0±200	13080	12829	236
AWI-5952.1.1	1130.5	*N. pachyderma*	11854±131	You et al., (24)	0±200	13186	13097	259
SUERC-47583	1140.5	*N. pachyderma*	12379±43	You et al., (24)	0±200	13777	13965	476
SUERC-51888	1150.5	*N. pachyderma*	12975±44	You et al., (24)	0±200	14650	14253	320
AWI-6488.1.1	1153.5	*N. pachyderma*	12942±102	You et al., (24)	0±200	14601	14342	312
AWI-6489.1.1	1155.5	*N. pachyderma*	12671±102	You et al., (24)	0±200	14212	14401	324
AWI-6490.1.1	1157.5	*N. pachyderma*	12518±106	You et al., (24)	0±200	13978	14459	345
AWI-6491.1.1	1159.5	*N. pachyderma*	12721±106	You et al., (24)	0±200	14288	14515	378
AWI-5954.1.1	1160.5	*N. pachyderma*	12544±120	You et al., (24)	0±200	14020	14656	374
AWI-6492.1.1	1162.5	*N. pachyderma*	12972±105	You et al., (24)	0±200	14643	15169	330
AWI-5955.1.1	1165.5	*N. pachyderma*	13965±135	You et al., (24)	0±200	16035	15943	315
AWI-5956.1.1	1167.5	*N. pachyderma*	14937±147	You et al., (24)	0±200	17265	16458	337
SUERC-47584	1170.5	*N. pachyderma*	15386±51	You et al., (24)	0±200	17797	17232	420

All AMS^{14}C dates were calibrated using the Marine 20 calibration curve (*75*). Modelled ages were obtained with the Bacon program (*76*). The 2σ uncertainties represent the 95% confidence intervals of the modelled ages. We applied a conservative reservoir correction uncertainty of $\Delta R = 0 \pm 200$ years for Core MSM12/2-05-01, which is broader than the $\Delta R = 0$ assumption commonly used in subpolar North Atlantic studies. Consequently, the age uncertainties (95% confidence ranges) remain relatively large and should be regarded as approximate.

Data S1. (separate file)

(1) HBI biomarker data (IP_{25}, dinosterol, and P_DIP_{25} values) from Core SL170, representing the last 16.82 to 10.09 ka BP.

(2) HBI biomarker data (IP_{25}, dinosterol, and P_DIP_{25} values) from Core MSM12/2-05-01, representing the last 18 to 9.8 ka BP

(3) Alkenone data (SST_U^K_{37}, $\%C_{37:4}$) from Core MSM12/2-01-01, representing the last 18 to 9.8 ka BP.
